# High feeding intensity increases the severity of fatty liver in the American mink (*Neovison vison*) with potential ameliorating role for long-chain n-3 polyunsaturated fatty acids

**DOI:** 10.1186/1751-0147-56-5

**Published:** 2014-01-16

**Authors:** Morag F Dick, Jennifer Hurford, Sha Lei, Anne-Mari Mustonen, Petteri Nieminen, Kirsti Rouvinen-Watt

**Affiliations:** 1 Department of Plant and Animal Sciences, Faculty of Agriculture, Dalhousie University, P.O. Box 550, B2N 5E3 Truro, NS, Canada; 2 Department of Bioscience and Biotechnology, Dalian University of Technology, 116024 Dalian, China; 3 Institute of Biomedicine/Anatomy, School of Medicine, Faculty of Health Sciences, University of Eastern Finland, P.O. Box 1627, FI-70211 Kuopio, Finland; 4 Department of Biology, Faculty of Science and Forestry, University of Eastern Finland, P.O. Box 111, FI-80101 Joensuu, Finland

**Keywords:** Fasting, Fatty liver, Mink, Obesity, Polyunsaturated fatty acids

## Abstract

**Background:**

Rapid body fat mobilization, obesity, and an inadequate supply of n-3 polyunsaturated fatty acids (PUFA) have been suggested to play roles in the etiology of fatty liver in the American mink (*Neovison vison*). This study examined the effects of feeding intensity and dietary fat source on fatty liver induced by fasting. In a multi-factorial design, 3 different fat sources (herring oil, rich in n-3 PUFA, soya oil, rich in n-6 PUFA, and canola oil, rich in n-9 monounsaturated fatty acids) were fed to mink at a low and high feeding intensity for 10 weeks, followed by an overnight or a 5-day fasting treatment to induce fatty liver.

**Results:**

Fasting led to the development of fatty liver with increased severity in the mink fed at the high feeding intensity. The herring oil diet, high in long-chain n-3 PUFA, was found to decrease the severity of fatty liver in the mink at the high feeding intensity.

**Conclusion:**

Preventing excessive weight gain and increasing dietary intake of n-3 long-chain PUFA may help prevent excessive lipid accumulation during prolonged periods of fasting or inappetence by promoting hepatic fatty acid oxidation.

## Background

Fatty liver syndrome is a common metabolic disease in farmed American mink (*Neovison vison*) and it has been reported to develop during acute stressful periods, when mink may show a loss in appetite for a few days [1]. Hepatic fatty infiltration is also a common pathological finding in female mink with nursing sickness [2]. Experimentally, food withdrawal has been used to model the development of the disease, with hepatic lipid accumulation occurring in less than 5 days of fasting [3]. Food deprivation can also result in borderline steatohepatitis in a closely related species, the European polecat (*Mustela putorius*) [4]. Studies using the mink model have shown that fat depots, particularly intra-abdominal, are readily mobilized during fasting and there is selective mobilization of n-3 polyunsaturated fatty acids (PUFA) from the adipose tissue and depletion in the liver [5]. This suggests that causative factors in mustelid fatty liver could be the rapid mobilization of body fat during fasting, obesity, and loss of n-3 PUFA, with similarities to human non-alcoholic fatty liver disease and feline hepatic lipidosis [3,4,6].

Excessive body fat is a common risk factor for fatty liver in both mink and cats (*Felis catus*) [1,7], and it is correlated with the severity of fatty liver in humans [8]. Increased adiposity in mink can lead to elevated blood glucose levels and the development of hyperinsulinemia [9]; derangements which, in humans, are associated with the severity of hepatic steatosis [10]. Furthermore, increased adiposity may increase circulating levels of free fatty acids (FFA) and subsequently the quantity of FFA the liver is exposed to [11,12].

The composition of dietary fats may also influence fatty liver, with an association of low proportions of n-3 PUFA or a high n-6:n-3 PUFA ratio with hepatic lipid accumulation [13]. The long-chain n-3 PUFA, 20:5n-3 and 22:6n-3, are suggested to be the most effective in preventing or ameliorating the development of fatty liver [14-17]. Within the liver, increasing long-chain n-3 PUFA may alter the balance between fatty acid oxidation and lipid secretion *vs. de novo* lipogenesis, and thereby reduce overall lipid accumulation [2]. This could make dietary fat an important consideration for mink as they are probably unable to sufficiently synthesize the long-chain n-3 and n-6 PUFA from their precursors [18].

Fatty liver disease in mink is initially asymptomatic and develops rapidly to the severe stages, thus the focus needs to be on preventative measures rather than treatment. Studies have examined the effect of diet on hepatic lipid content under normal physiological conditions [18] and the relationship between body fat and hepatic lipid content in fasted mink [19], but neither has been investigated directly during the development of fatty liver. The aim of this study was to examine how feeding intensity (FI) and dietary fat sources varying in n-3 and n-6 PUFA affect the development of fasting-induced fatty liver in mink.

## Methods

Seventy-two standard black five-month-old mink (36 male, 36 female) were used at the Canadian Centre for Fur Animal Research (Truro, NS). The mink were housed individually in pens (W × H × L: 30 cm × 45 cm × 75 cm) with wood chip-bedded nest boxes. The study was carried out in accordance with the Canadian Council on Animal Care [20] and approval of the Animal Care and Use Committee of the Dalhousie University, Faculty of Agriculture (formerly Nova Scotia Agricultural College, Truro; Permit ACUC#2007-041).

In this multi-factorial study, each mink was assigned to an experimental diet containing herring oil (HO), soya oil (SO), or canola oil (CO) as the dietary fat source (Tables 1 and 2), and the diets were formulated to meet the nutritional recommendations for mink [21]. Fatty acid profiles of the diets were analyzed using gas–liquid chromatography (GC-FID and GC-MS, 6890 N network GC-system, Agilent Technologies Inc., Palo Alto, CA) [22]. Each diet was fed either at a low feeding intensity (LFI) of 80% recommended dietary allowance (RDA) or a high feeding intensity (HFI) of 120% RDA, with the RDA adjusted for sex, month, and metabolic body weight (BW^0.75^ kg) [21]. BW and feed intake were measured monthly over a three-day period by determining the amount of feed left uneaten.

**Table 1:** Ingredient formulation of the herring oil, soya oil, and canola oil experimental diets

	**Diet**		
**Ingredient, *g/kg***	**Herring oil**	**Soya oil**	**Canola oil**
Cod racks	525	525	525
Pork liver	135	135	135
Pork spleen	70	70	70
Extruded barley	144	144	144
Corn gluten meal	31	31	31
Herring oil	47	-	-
Soya oil	-	47	-
Canola oil	-	-	47
Vitamin mineral premix^a^	4	4	4
Water	44	44	44

^a^Vitamin Mineral Premix growing/furring [(concentration/purity), amount per tonne of premix]: vitamin A (600 × 10^5^ IU/kg), 600 IU; vitamin D (500 × 10^5^ IU/kg), 600 IU; vitamin E (500 000 IU/kg), 150 IU; vitamin K (33%), 0.66 g; vitamin C (97.5%), 94.6 g; riboflavin (95%), 47.5 g; DL Ca-pantothenate (45%), 6.30 g; niacin (99%), 19.8 g; vitamin B_12_ (1000 mg/kg), 0.02 g; folic acid (3%), 0.45 g; biotin (2%), 0.1 g; choline chloride (60%), 600 g; thiamine (97 × 10^4^ mg/kg), 3.88 g; pyridoxine (99 × 10^4^ mg/kg), 4.95 g; manganous oxide (60%), 10.8 g; zinc oxide (80%), 30.4 g; cupric sulphate (25%), 5 g; cobalt carbonate (45%), 0.45 g; selenium premix (675 mg/kg), 0.22 g; ferrous sulphate (27%), 99.9 g; ground limestone (38%), 190 g; ethoxyquin (50%), 45 g; wheat middlings 3324 g.

**Table 2:** Nutrient composition and fatty acid profiles of the herring oil, soya oil, and canola oil experimental diets

		**Experimental diet**		
		**Herring oil**	**Soya oil**	**Canola oil**
Composition	n	3	3	3
*(as fed)*	Dry Matter, %	39.1 ± 0.7	39.8 ± 0.9	39.5 ± 0.5
	Crude Protein, %	15.1 ± 0.3	15.6 ± 0.5	14.8 ± 0.3
	Crude Fat, %	6.7 ± 0.4	7.7 ± 0.9	6.5 ± 0.5
	Carbohydrate, %	12.5 ± 0.8	13.5 ± 1.2	13.86 ± 0.5
	Ash, %	4.0 ± 0.2	3.8 ± 0.2	3.8 ± 0.3
	ME^1^, *kJ/kg*	6364 ± 100	6904 ± 301	6414 ± 117
Fatty acid profile,	Total SFA	25.7 ± 0.5^A^	20.4 ± 0.4^B^	14.9 ± 0.5^C^
*molar%*	18:1n-9 + 11	10.6 ± 0.5^C^	17.4 ± 0.4^B^	48.3 ± 0.5^A^
	22:1n-11	17.34 ± 0.05^A^	0.13 ± 0.03^B^	0.08 ± 0.01^C^
	Total MUFA	53.17 ± 0.29^A^	20.99 ± 0.29^B^	53.85 ± 0.29^A^
	18:2n-6	8.86 ± 0.08^C^	45.98 ± 0.08^A^	21.18 ± 0.02^B^
	20:4n-6	1.47 ± 0.13	1.23 ± 0.13	1.08 ± 0.13
	Total n-6 PUFA	10.83 ± 0.55^C^	47.56 ± 0.55^A^	22.61 ± 0.55^B^
	18:3n-3	0.75 ± 0.14^C^	8.12 ±0.14^A^	6.65 ± 0.14^B^
	20:5n-3	4.05 ± 0.07^A^	0.82 ± 0.07^B^	0.54 ± 0.07^C^
	22:6n-3	3.40 ± 0.18^A^	1.52 ± 0.18^B^	1.03 ± 0.18^B^
	Total n-3 PUFA	10.29 ± 0.29^A^	10.97 ± 0.29^A^	8.57 ± 0.29^B^
	n-6:n-3 PUFA	1.05 ± 0.03^C^	4.35 ± 0.03^A^	2.63 ± 0.03^B^

Values are lsmeans ± SEM. Labeled means with no common letters differ within row (P ≤ 0.05). Metabolizable energy, ME; monounsaturated fatty acids, MUFA; polyunsaturated fatty acids, PUFA; saturated fatty acids, SFA.

^1^ME was calculated as the sum of each of the energy nutrients multiplied by their digestibility coefficients and energy values [(crude protein × 0.85 × 18.8 kJ/g) + (crude fat × 0.90 × 39.8 kJ/g) + (carbohydrate × 0.70 × 17.6 kJ/g)].

After feeding the dietary regimes for 10 weeks, the mink were divided further into two groups, the control group which was fasted overnight and the fasted group which was deprived of food for a 5-day period. Following the fasting treatments, the mink were anaesthetized with intramuscular injection of xylazine (3.4 mg/kg BW) and ketamine hydrochloride (8.5 mg/kg BW). A blood sample was taken by cardiac puncture (Vacutainer® EDTA tubes) and the mink were killed using an intra-cardiac injection of pentobarbital sodium (106 mg/kg BW). Immediately following euthanasia, a liver sample was flash frozen in liquid nitrogen and stored at −80°C for later lipid extraction using the Folch method [23]. Hepato-somatic index (HSI) was calculated as follows: (liver weight, g/BW, g) × 100.

During the feeding trial, blood glucose measurements were taken postprandially using the Accu-Check Compact™ blood glucose monitor (Roche Diagnostics, Laval, QC). Clinical chemistry analyses were carried out on plasma (stored at −80°C) for urea, glucose, alanine transaminase (ALT), and alkaline phosphatase (AKPH) at the Diagnostic Services Laboratory of the Atlantic Veterinary College (Charlottetown, PEI). Further analyses were performed at the University of Eastern Finland (Joensuu, Finland) for plasma levels of triacylglycerols (TAG), low-density lipoprotein (LDL) and high-density lipoprotein (HDL) cholesterol, and FFA as outlined by Mustonen *et al*. [5]. Plasma concentrations of insulin, leptin, triiodothyronine (T_3_), and thyroxine (T_4_) were determined using established validated methodology according to Mustonen *et al*. [24].

Statistical analysis was carried out using the Proc Mixed in SAS *v*.9 (SAS Institute Inc., Cary, NC) to determine significant effects and interactions, and significant differences within these interactions. The monthly BW, feed intake, and blood glucose levels were analyzed using a split-plot design, with the repeated measures option. A split-split-plot design was used for the final blood analyses. To simplify analysis, in cases of a 3-way interaction that included fasting as an effect or a 4-way interaction, comparisons were made within the control and fasted mink, and between the individual control and fasted treatments only. The Pearson’s correlation coefficients (r_p_) were calculated using SAS *v.*9. P ≤ 0.05 was considered statistically significant and the P-values are included in the online additional documentation (Additional files 1, 2 and 3). Results are presented as lsmeans ± SEM.

## Results

### Feed intake and body weights

Feed intake was higher in the males compared to the females (1452 ± 25 kJ/day and 84.1 ± 1.5 g DM/day *vs.* 1038 ± 25 kJ/day and 60.0 ± 1.5 g DM/day). The mink fed at the LFI had a constant feed intake of 80% RDA throughout the trial, while intake increased in the HFI group over time, reaching 103% RDA at the end of the trial (Table 3). Diet also influenced feed intake, with increased caloric consumption from October to November only in the HO group.

**Table 3:** Feed intake of the mink fed at the LFI and HFI and fed the herring oil, soya oil, and canola oil experimental diets during the feeding trial period

	**Feed intake, *kJ/day***		**Feed intake, % *RDA***	
	**Feeding intensity**		**Feeding intensity**	
	**LFI**	**HFI**	**LFI**	**HFI**
October	1147 ± 33^Ab^	1223 ± 37^Ba^	80 ± 2^Ab^	89 ± 2^Ba^
November	1114 ± 29^Ab^	1495 ± 29^Aa^	80 ± 1^Ab^	103 ± 1^Aa^
	**Feed intake, *kJ/day***			
	**Diet**			
	**Herring oil**	**Soya oil**	**Canola oil**	
October	1193 ± 45^B^	1196 ± 45	1164 ± 45	
November	1461 ± 36^Aa^	1285 ± 36^b^	1160 ± 36^c^	

Values are lsmeans ± SEM. Labeled means with no common capital letters differ within the column, and means with no common lowercase letters differ within the row (P ≤ 0.05). High feeding intensity, HFI; low feeding intensity, LFI; recommended dietary allowance, RDA.

During the feeding trial, both sexes lost weight when fed at the LFI, however, in the HFI group, only males exhibited significant weight gain (Table 4). The feeding trial resulted in two distinct BW groups in both sexes. These BW distinctions remained after the fasting period and at final sampling. The difference in the final BW revealed the fasted mink to be generally lighter than the controls, with heavier males in the HO group.

**Table 4:** Mink body weight during the feeding trial, at the end of the fasting period, and final body weight and liver weight at sampling

		***Body weight during feeding trial, g***					
		**Sex × feeding intensity interaction**					
		**Male**			**Female**		
		**LFI**	**HFI**		**LFI**	**HFI**	
	Sep 27	2381 ± 47^A^	2376 ± 47^B^		1271 ± 47^A^	1287 ± 47	
	Oct 25	2283 ± 47^B*^	2511 ± 47^A^		1202 ± 47^B*^	1326 ± 47	
	Nov 22	2072 ± 47^C*^	2528 ± 47^A^		1096 ± 47^C*^	1324 ± 47	
		***Body weights during the 5-day fasting period, g***					
		**Feeding intensity**			**Sex**		
		**LFI**	**HFI**		**Male**	**Female**	
	24-hour fasting	1411 ± 64^C^	1851 ± 64^A^		2153 ± 75^A^	1108 ± 75^C^	
	5-day fasting	1211 ± 64^D^	1640 ± 64^B^		1902 ± 75^B^	949 ± 75^D^	
		***Final weight at sampling, g***					
		**Sex × feeding intensity interaction**					
		**Male**			**Female**		
		**LFI**	**HFI**		**LFI**	**HFI**	
	Body weight	1770 ± 78^B^	2338 ± 78^A^		909 ± 78^D^	1168 ± 78^C^	
	Liver weight	41.9 ± 1.7^B^	55.1 ± 1.7^A^		25.2 ± 1.7^D^	30.9 ± 1.7^C^	
		**Sex × diet interaction**					
		**Male**			**Female**		
		**Herring oil**	**Soya oil**	**Canola oil**	**Herring oil**	**Soya oil**	**Canola oil**
Body weight	Control	2504 ± 106^A*^	2117 ± 106^B^	1999 ± 106^B^	1085 ± 106^C^	1155 ± 106^C^	1143 ± 106^C^
	Fasted	1867 ± 106^A^	1884 ± 106^A^	1954 ± 106^A^	1025 ± 106^B^	928 ± 106^B^	895 ± 106^B^
Liver weight	Control	54.7 ± 2.4^A*^	45.6 ± 2.4^B^	44.8 ± 2.4^B^	27.6 ± 2.4^C^	27.4 ± 2.4^C^	28.7 ± 2.4^C^
	Fasted	47.0 ± 2.4^A^	48.3 ± 2.4^A^	49.5 ± 2.4^A^	30.4 ± 2.4^B^	27.3 ± 2.4^B^	26.8 ± 2.4^B^

Values are lsmeans ± SEM. For feeding trial weights, labeled means with no common letters differ within sex and feeding intensity, and means with * differ between feeding intensity within sex (P ≤ 0.05). For 5-day fasting group weights, labeled means with no common letters differ within treatment response. For final body and liver weights, labeled means with no common letters differ within fasting treatments, and means with * differ between fasting treatments. High feeding intensity, HFI; low feeding intensity, LFI.

### Liver health

Liver lipid% was higher in the fasted mink, and it was the highest in the mink fed at the HFI (Table 5). A significant interaction between diet and FI was observed, with the highest liver lipid levels in the mink fed the SO and CO diets at the HFI, and the lowest in the mink fed the SO diet at the LFI, which did not differ significantly from the HO groups (Table 6). Liver weights were higher in the males than females, and within sex higher in the HFI groups. No differences were observed in the females with regard to diet or fasting in liver weight; however, in the males, the heaviest livers were found in the control HO group, and their average liver weights were higher than those of the fasted HO group and the control SO and CO mink. HSI was greater in the females (females: 2.76 ± 0.06, males: 2.38 ± 0.06), and an overall higher HSI was observed in the fasted mink (control: 2.37 ± 0.08, fasted: 2.77 ± 0.08). The mink of the LFI HO group had the highest HSI, which did not differ from the HFI SO group or the LFI and HFI CO groups.

**Table 5:** Effect of fasting and feeding intensity on liver lipid%, plasma glucose, AKPH, and LDL-cholesterol

	**Control**		**Fasted**	
	**LFI**	**HFI**	**LFI**	**HFI**
Liver Lipids, %	5.9 ± 1.1^C^	6.6 ± 1.1^C^	16.2 ± 1.1^B^	21.7 ± 1.1^A^
Glucose, *mmol/L*	8.3 ± 0.3^A^	8.5 ± 0.3^A^	6.5 ± 0.3^C^	7.6 ± 0.3^B^
AKPH, *U/L*	22 ± 3^AB^	21 ± 3^AB^	29 ± 3^A^	17 ± 3^B^
LDL-Cholesterol, *mmol/L*	0.58 ± 0.03^A^	0.41 ± 0.03^B^	0.28 ± 0.03^C^	0.26 ± 0.03^C^

Values are lsmeans ± SEM. Labeled means with no common letters differ within the response variable (P ≤ 0.05). Alkaline phosphatase, AKPH; high feeding intensity, HFI; low-density lipoprotein, LDL; low feeding intensity, LFI.

**Table 6:** Effect of the herring oil, soya oil, and canola oil experimental diets and feeding intensity on mink liver lipid%, liver weight, and HSI

	**LFI**			**HFI**		
	**Herring oil**	**Soya oil**	**Canola oil**	**Herring oil**	**Soya oil**	**Canola oil**
Liver Lipids, %	11.7 ± 1.3^BC^	8.9 ± 1.3^C^	12.7 ± 1.3^AB^	12.0 ± 1.3^BC^	15.7 ± 1.3^A^	14.7 ± 1.3^A^
Liver Weight, *g*	37.0 ± 1.7^B^	30.4 ± 1.7^C^	33.2 ± 1.7^BC^	42.9 ± 1.7^A^	44.4 ± 1.7^A^	41.7 ± 1.7^A^
HSI	2.74 ± 0.09^A^	2.42 ± 0.09^C^	2.70 ± 0.09^AB^	2.43 ± 0.09^BC^	2.61 ± 0.09^ABC^	2.52 ± 0.09^ABC^

Values are lsmeans ± SEM. Labeled means with no common letters differ within the response variable (P ≤ 0.05). Hepato-somatic index, HSI; high feeding intensity, HFI; low feeding intensity, LFI.

### Clinical chemistry and endocrinology

Blood glucose levels were the highest at the start of the feeding trial (September: 4.6 ± 0.1, October: 3.8 ± 0.1, November 3.7 ± 0.1 mmol/L). The final glucose concentrations, analyzed on plasma, were higher in the control mink, and within the fasted mink they were higher in the HFI group (Table 5). The fasted mink had higher plasma ALT activities (control: 139 ± 22, fasted: 299 ± 22 U/L). Plasma AKPH activities were higher in the fasted mink fed at the LFI compared to the HFI, but did not differ from the controls.

In the control groups, LDL-cholesterol was higher in the mink fed at the LFI than the HFI, and the levels were lower in the fasted mink where no effect of FI was found (Table 5). HDL-cholesterol was lower in the fasted mink (3.64 ± 0.08 *vs.* 3.32 ± 0.08 mmol/L), and the levels were also affected by FI and diet; the highest HDL-cholesterol levels were in the mink fed the SO diet at the LFI, with no differences among the other groups (Table 7). LDL- and HDL-cholesterol both showed a significant diet and sex interaction, with LDL-cholesterol being higher in the males fed the CO diet and the females fed the HO diet. The highest HDL-cholesterol levels were observed in the females fed the SO diet and the males fed the CO diet, and for both sexes the lowest concentrations were observed when fed the HO diet. Plasma FFA levels did not differ among the controls and were higher in the fasted mink, with the highest levels observed in the fasted LFI CO group (Table 8). Higher TAG levels were noted in the fasted mink, with the exception of the mink fed the SO diet at the HFI. Additionally, the males had lower TAG levels than the females (0.94 ± 0.04 *vs.* 1.13 ± 0.04 mmol/L).

**Table 7:** Effect of feeding intensity, sex, and the herring oil, soya oil, and canola oil experimental diets on plasma LDL- and HDL-cholesterol levels

	**Feeding intensity × diet interaction**					
	**LFI**			**HFI**		
	**Herring oil**	**Soya oil**	**Canola oil**	**Herring oil**	**Soya oil**	**Canola oil**
HDL-Cholesterol, *mmol/L*	3.35 ± 0.12^B^	4.05 ± 0.12^A^	3.52 ± 0.12^B^	3.13 ± 0.12^B^	3.22 ± 0.12^B^	3.51 ± 0.12^B^
	**Sex × diet interaction**					
	**Male**			**Female**		
	**Herring oil**	**Soya oil**	**Canola oil**	**Herring oil**	**Soya oil**	**Canola oil**
LDL-Cholesterol, *mmol/L*	0.35 ± 0.04^AB^	0.34 ± 0.04^B^	0.44 ± 0.04^A^	0.44 ± 0.04^A^	0.39 ± 0.04^AB^	0.34 ± 0.04^B^
HDL-Cholesterol, *mmol/L*	2.97 ± 0.13^D^	3.27 ± 0.13^CD^	3.45 ± 0.13^BC^	3.51 ± 0.13^BC^	4.01 ± 0.13^A^	3.58 ± 0.13^B^

Values are lsmeans ± SEM. Labeled means with no common letters differ within the response variable (P ≤ 0.05). High-density lipoprotein, HDL; high feeding intensity, HFI; low-density lipoprotein, LDL; low feeding intensity, LFI.

**Table 8:** Effect of the herring oil, soya oil, and canola oil experimental diets, LFI and HFI, and fasting on mink plasma levels of FFA and TAG

		**FFA, *mmol/L***		**TAG, *mmol/L***	
		**Control**	**Fasted**	**Control**	**Fasted**
Herring oil	LFI	0.50 ± 0.14^*^	1.32 ± 0.14^ABC^	0.94 ± 0.09^AB*^	1.31 ± 0.09^A^
	HFI	0.58 ± 0.14	0.95 ± 0.14^C^	0.89 ± 0.0^AB*^	1.34 ± 0.09^A^
Soya oil	LFI	0.44 ± 0.14^*^	0.96 ± 0.14^C^	0.72 ± 0.09^B*^	1.15 ± 0.09^AB^
	HFI	0.46 ± 0.14^*^	1.52 ± 0.14^AB^	1.06 ± 0.09^A^	1.02 ± 0.09^B^
Canola oil	LFI	0.60 ± 0.14^*^	1.63 ± 0.14^A^	0.76 ± 0.09^B*^	1.11 ± 0.09^AB^
	HFI	0.57 ± 0.14^*^	1.10 ± 0.14^ABC^	0.85 ± 0.09^AB*^	1.25 ± 0.09^AB^

Values are lsmeans ± SEM. Labeled means with no common letters differ within the response variable column, and means with *differ within the response variable row (P ≤ 0.05). Free fatty acids, FFA; high feeding intensity, HFI; low feeding intensity, LFI; triacylglycerols, TAG.

Insulin levels were lower in the males than females (4.32 ± 0.35 *vs.* 5.98 ± 0.35 μU/mL), the mink fed at the HFI had higher insulin levels than those fed at the LFI (5.67 ± 0.36 *vs.* 4.63 ± 0.36 μU/mL), and the concentrations correlated significantly with the body fat% (%BF; r_p_ = 0.26, P = 0.028). Overall, the fasted mink had lower insulin levels (4.34 ± 0.36 *vs.* 5.95 ± 0.36 μU/mL). Leptin levels in the control mink were higher when fed at the HFI (3.41 ± 0.19 ng/mL) compared to the LFI (1.91 ± 0.19 ng/mL) and correlated with %BF (r_p_ = 0.58, P = 0.001). In the fasted mink, leptin levels were lower than in the controls and did not differ between the FI groups (LFI: 1.25 ± 0.19, HFI: 1.36 ± 0.19 ng/mL). T_3_ tended to be higher in the mink fed at the HFI, correlated with %BF (r_p_ = 0.60, P = 0.001), and was lower in the fasted mink (Table 9). T_4_ did not correlate with %BF (r_p_ = 0.36, P = 0.77). T_4_ levels differed significantly depending on the FI of the HO mink (LFI: 10.2 ± 0.8, HFI: 13.1 ± 0.8 nmol/L) but there were no other significant differences within diets or FI (SO; LFI: 11.7 ± 0.8, HFI: 10.0 ± 0.8, CO; LFI: 11.8 ± 0.8, HFI: 10.6 ± 0.8 nmol/L).

**Table 9:** **Effect of fasting and feeding intensity combined with the herring oil, soya oil, and canola oil experimental diets on T**_**3 **_**concentrations**

		**T**_**3**_**, *nmol/L***			
		**Fasting treatment**		**Feeding intensity**	
		**Control**	**Fasted**	**LFI**	**HFI**
Herring oil	Male	0.81 ± 0.05^C*^	0.65 ± 0.05^C^	0.65 ± 0.06^B*^	0.82 ± 0.06^C^
	Female	0.98 ± 0.05^AB*^	0.85 ± 0.06^AB^	0.73 ± 0.06^AB*^	1.10 ± 0.06^A^
Soya oil	Male	0.86 ± 0.05^BC*^	0.70 ± 0.05^BC^	0.65 ± 0.06^B*^	0.91 ± 0.06^BC^
	Female	1.07 ± 0.05^A*^	0.71 ± 0.06^BC^	0.85 ± 0.06^A^	0.94 ± 0.06^BC^
Canola oil	Male	0.83 ± 0.05^C*^	0.70 ± 0.05^BC^	0.69 ± 0.06^B*^	0.84 ± 0.06^C^
	Female	0.92 ± 0.05^BC^	0.93 ± 0.07^A^	0.78 ± 0.06^AB*^	1.07 ± 0.07^AB^

Values are lsmeans ± SEM. Labeled means with no common letters differ within the response variable column and means with *differ within the response variable row (P ≤ 0.05). High feeding intensity, HFI; low feeding intensity, LFI; triiodothyronine, T_3_.

## Discussion

By modifying the FI, we created two distinct BW groups of lighter and heavier mink. Dietary fat source also influenced the BW of the mink, as observed previously by Käkelä *et al*. [18]. According to our results, the control males fed the HO diet were significantly heavier than the males fed the SO or CO diets. This difference was possibly caused by the increased feed intake in the HO males.

Fasting expectedly caused weight loss and it is known to result in the development of fatty liver in carnivores [3-5,25]. A 5% liver TAG threshold is used for the diagnosis of fatty liver in cats [26]. Using this threshold, the results of previous studies examining both hepatic TAG and total lipid levels in mink suggest that the corresponding total liver lipid level threshold for fatty liver in mink would be between 9 to 12% [3,18]. Based on this threshold range, the control mink appeared to have healthy hepatic lipid levels. With fasting, hepatic lipid levels increased, exceeding 12%, along with plasma ALT activities, an indicator of liver dysfunction [27]. Higher liver lipid values in the mink fed at the HFI compared to the LFI revealed the negative implications of excessive adiposity on liver health. The severity of the hepatic lipid accumulation in the mink fed at the HFI was lower when the mink were fed the HO diet compared to the SO and CO diets, suggesting that n-3 long-chain PUFA may help ameliorate or slow the development of fatty liver in over-conditioned mink. The HSI results were not in agreement with the liver lipid%, unlike in a previous study [3]. Caution is needed when using HSI, as in the current study it was possible for lean mink without fatty liver to have higher HSI values than heavier mink with fatty liver.

Fasting causes lipolysis in body fat depots releasing fatty acids, and the liver may increase its fatty acid uptake from the blood in proportion to their concentration in circulation [12]. Plasma FFA levels were not affected by dietary treatment in the controls. Similar to previous studies [3,5], the FFA and TAG levels increased with fasting and the FFA levels were higher in the mink with higher liver lipid levels, with the exception of the mink fed the HO diet at the LFI. These results reflect the rapid mobilization of the body fat reserves [3], and are also observed in clinical cases of feline hepatic lipidosis [28]. An exception to this was the mink fed the SO diet at the HFI, where the high dietary n-6:n-3 PUFA ratio may decrease the liver secretion of TAG [29], contributing to the increased liver lipid content in these mink. The decreased secretion may also explain the lack of response to fasting compared to the other groups.

Plasma LDL-cholesterol levels were lower in the fasted and HFI groups, differing from Mustonen *et al*. [5] where fasting did not have an effect. Ibrahim *et al*. [30] reported increased LDL-cholesterol levels in cats with diet-induced obesity. No clear explanations are apparent for these differences in LDL-cholesterol. Decreases in HDL-cholesterol in the fasted mink are in agreement with Mustonen *et al*. [5], and could be related to increased clearance by the liver [30]. Whether this is a result of fasting or a consequence of fatty liver is not known, and no changes were evident in clinical cases of feline hepatic lipidosis [28].

Insulin resistance and hyperglycemia are associated with fatty liver, with the development of the two being able to further worsen each other [10]. Monthly blood glucose concentrations were within the normal range for mink [31], which differs from a previous study where high FI was associated with hyperglycemia [9]. The final plasma glucose values were elevated slightly in the control mink, but the results are difficult to interpret as handling stress and ketamine can also increase the blood glucose levels [32]. In the fasting model, mink maintain normoglycemia [5], and do not develop the severe derangement of glucose regulation that can be observed in nursing sickness in mink [33] and in feline hepatic lipidosis [28]. Insulin concentrations were not elevated either, and decreased during fasting, helping to spare glucose and increase fat mobilization [3,24]. Although the mink appear to have remained normoglycemic throughout the study, the positive correlation between insulin and %BF suggests that long-term monitoring of blood glucose concentrations may be interesting as there is evidence for impaired glycemic control in over-conditioned mink [9].

Under normal physiological conditions, plasma leptin levels in mink increase with increasing BW [34], and decrease during fasting [3,24]. This relationship between body fat and leptin levels was maintained in the control mink. However, the effect of body condition on leptin levels was not present in the fasted mink, with similar levels in the LFI and HFI groups, indicating that fasting has a stronger influence on leptin levels than adiposity. Although higher leptin levels are observed in clinical cases of feline hepatic lipidosis [28], they are likely to be related to increased adiposity. Regarding the T_3_ concentrations, a clear decrease was observed in the fasted mink. This was documented also previously [24] and probably represents an adaptation of energy saving.

The balance of lipid delivery and metabolism in the liver contributes to the net hepatic lipid accumulation. Obesity may aggravate the problem by increasing the amount of fat available for mobilization. Dietary fat may also influence lipid metabolism in the liver, with long-chain n-3 PUFA promoting hepatic lipid excretion and oxidation [29]. Ahlstrøm and Skrede [35] found a positive effect of fish oil, high in long-chain n-3 PUFA, on hepatic metabolism, with dietary levels correlating positively to liver peroxisomal *β*-oxidation activity. During periods of hepatic lipid accumulation, it would be beneficial to be able to increase fatty acid oxidation within the liver. This may help to explain why within the over-conditioned HFI group, the mink fed the HO diet had the lowest liver lipid levels. In addition, the beneficial role of HO could potentially be attributed to the higher levels of long-chain monounsaturated fatty acids, also shown to increase oxidative capacity of liver and muscle [36]. A longer-term controlled feeding experiment, accounting for the seasonal fluctuations in body condition, with a larger number of mink per sub-treatment, would be necessary to confirm the potential beneficial effects of dietary long-chain n-3 PUFA on liver health.

Despite the significant hepatic lipid accumulation as a result of fasting, the mink appeared alert and healthy. It remains unknown what can cause the relatively benign lipid accumulation to progress rapidly to the life-threatening stage in hypercarnivores, such as mink and cats [1,6]. Currently there are also no specific pharmaceutical therapies available. It is therefore evident that preventing the over-feeding and rapid slimming of mink throughout the year may help maintain liver health. It is noteworthy that the current RDA guidelines, used in this study [21], may exceed the mink’s energy requirements leading to obesity. The HFI mink consumed just over 100% RDA of what was offered but were still over-conditioned.

To conclude, by feeding the mink different dietary fat sources at the LFI and HFI we were able to examine the effects of FI and fatty acid nutrition on the development of fasting-induced fatty liver. FI had no effect on the hepatic lipid content of the control mink, whereas fasting caused the development of fatty liver in the mink with increasing severity in those fed at the HFI. Preventing over-conditioning and increasing dietary intake of long-chain n-3 PUFA were found to be beneficial in decreasing the severity of lipid accumulation.

## Abbreviations

ALT: Alanine transaminase; AKPH: Alkaline phosphatase; %BF: Body fat%; BW: Body weight; CO: Canola oil; FI: Feeding intensity; FFA: Free fatty acid; HSI: Hepato-somatic index; HO: Herring oil; HDL: High-density lipoprotein; HFI: High feeding intensity; LDL: Low-density lipoprotein; LFI: Low feeding intensity; PUFA: Polyunsaturated fatty acid; RDA: Recommended dietary allowance; SO: Soya oil; T4: Thyroxine; TAG: Triacylglycerols; T3: Triiodothyronine.

## Competing interests

The authors declare that they have no competing interests.

## Authors’ contributions

MFD, A-MM, PN, KR-W designed the research. MFD, JH, SL, A-MM, PN, KR-W conducted research. MFD analyzed data and led the writing of the paper. MFD and KR-W share primary responsibility for final content. All authors read and approved the final manuscript.

## Supplementary Material

Additional file 1 P-values of the main effects and interactions for mink body weights, feed intake, and blood glucose during the feeding trial from September to November. Click here for file

Additional file 2 P-values of the main effects and interactions for final body weight and liver responses. Click here for file

Additional file 3 P-values of the main effects and interactions for the mink clinical chemistry and endocrinological responses. Click here for file

## References

[B1] Hunter B Lemieux N Mink … Biology, Health and Disease 1996 Guelph, Canada: University of Guelph Graphic and Print Services

[B2] Rouvinen-Watt K Nursing sickness in the mink – a metabolic mystery or a familiar foe? Can J Vet Res 2003 56 161 168 12889720 PMC227047

[B3] Rouvinen-Watt K Mustonen A-M Conway R Pal C Harris L Saarela S Strandberg U Nieminen P Rapid development of fasting-induced hepatic lipidosis in the American mink (*Neovison vison*): Effects of food deprivation and re-alimentation on body fat depots, tissue fatty acid profiles, hematology and endocrinology Lipids 2010 56 111 128 10.1007/s11745-009-3377-4 20020218

[B4] Nieminen P Mustonen A-M Kärjä V Asikainen J Rouvinen-Watt K Fatty acid composition and development of hepatic lipidosis during food deprivation—mustelids as a potential animal model for liver steatosis Exp Biol Med 2009 56 278 286 10.3181/0806-RM-210 19144866

[B5] Mustonen A-M Pyykönen T Paakkonen T Ryökkynen A Asikainen J Aho J Mononen J Nieminen P Adaptations to fasting in the American mink (*Mustela vison*): carbohydrate and lipid metabolism Comp Biochem Physiol A 2005 56 195 202 10.1016/j.cbpb.2004.12.004 15748859

[B6] Armstrong PJ Blanchard G Hepatic lipidosis in cats Vet Clin North Am Small Anim Pract 2009 56 599 616 10.1016/j.cvsm.2009.03.003 19524794

[B7] Dimski DS Taboada J Feline idiopathic hepatic lipidosis Vet Clin North Am Small Anim Pract 1995 56 357 373 7785168 10.1016/s0195-5616(95)50031-2

[B8] York LW Puthalapattu S Wu GY Nonalcoholic fatty liver disease and low-carbohydrate diets Annu Rev Nutr 2009 56 365 379 10.1146/annurev-nutr-070208-114232 19575599

[B9] Rouvinen-Watt K Murphy J Chan C Effect of feeding intensity on body condition and glycemic control in mink *Mustela vison* Scientifur 2004 56 129 135

[B10] Marceau P Biron S Hould F-S Marceau S Simard S Thung SN Kral JG Liver pathology and the metabolic syndrome X in severe obesity J Clin Endocrinol Metab 1999 56 1513 1517 10323371 10.1210/jcem.84.5.5661

[B11] Jensen MD Haymond MW Rizza RA Cryer PE Miles JM Influence of body fat distribution on free fatty acid metabolism in obesity J Clin Invest 1989 56 1168 1173 10.1172/JCI113997 2649512 PMC303803

[B12] Bradbury MW Lipid metabolism and liver inflammation. I. Hepatic fatty acid uptake: possible role in steatosis Am J Physiol Gastrointest Liver Physiol 2006 56 G194 G198 10.1152/ajpgi.00413.2005 16407588

[B13] Araya J Rodrigo R Videla L Thielemann L Orellana M Pettinelli P Poniachik J Increase in long-chain polyunsaturated fatty acid n-6/n-3 ratio in relation to hepatic steatosis in patients with non-alcoholic fatty liver disease Clin Sci 2004 56 635 643 10.1042/CS20030326 14720121

[B14] Lindén D Lindberg K Oscarsson J Claesson C Asp L Li L Gustafsson M Borén J Olofsson S-O Influence of peroxisome proliferator-activated receptor α agonists on the intracellular turnover and secretion of apolipoprotein (Apo) B-100 and ApoB-48 J Biol Chem 2002 56 23044 23053 10.1074/jbc.M110416200 11925428

[B15] Levy JR Clore JN Steven W Dietary n-3 polyunsaturated fatty acids decrease hepatic triglycerides in Fischer 344 rats Hepatology 2004 56 608 616 14999679 10.1002/hep.20093

[B16] Alwayn IPJ Gura K Nosé V Zausche B Javid P Garza J Verbesey J Voss S Ollero M Andersson C Bistrian B Folkman J Puder M Omega-3 fatty acid supplementation prevents hepatic steatosis in a murine model of nonalcoholic fatty liver disease Pediatr Res 2005 56 445 452 10.1203/01.PDR.0000153672.43030.75 15659701

[B17] Capanni M Calella F Biagini MR Genise S Raimondi L Bedogni G Svegliati-Baroni G Sofi F Milani S Abbate R Surrenti C Casini A Prolonged n-3 polyunsaturated fatty acid supplementation ameliorates hepatic steatosis in patients with non-alcoholic fatty liver disease: a pilot study Aliment Pharmacol Ther 2006 56 1143 1151 10.1111/j.1365-2036.2006.02885.x 16611275

[B18] Käkelä R Pölönen I Miettinen M Asikainen J Effects of different fat supplements on growth and hepatic lipids and fatty acids in male mink Acta Agric Scand Sect A, Animal Sci 2001 56 217 223

[B19] Clausen TN Sandbøl P Fasting of male mink after mating. Influence on liver fat and blood ketone content 2006 Akureyri, Iceland: Proceedings of the Nordic Association of Agricultural Scientists Seminar

[B20] Canadian Council on Animal Care Guide to the Care and Use of Experimental Animals 1993 Ottawa, Canada: Canadian Council on Animal Care

[B21] Rouvinen-Watt K White MB Campbell R Mink Feeds and Feeding 2005 Ottawa, Canada: Ontario Ministry of Agriculture and Food and Truro, Canada: Nova Scotia Agricultural College

[B22] Nieminen P Käkelä R Pyykönen T Mustonen A-M Selective fatty acid mobilization in the American mink (*Mustela vison*) during food deprivation Comp Biochem Physiol B 2006 56 81 93 10.1016/j.cbpb.2006.06.007 16899381

[B23] Folch J Lees M Sloane Stanley GH A simple method for the isolation and purification of total lipides from animal tissues J Biol Chem 1957 56 497 509 13428781

[B24] Mustonen A-M Saarela S Pyykönen T Nieminen P Endocrinologic adaptations to wintertime fasting in the male American mink (*Mustela vison*) Exp Biol Med 2005 56 612 620 10.1177/153537020523000903 16179729

[B25] Szabo J Ibrahim WH Sunvold GD Dickey KM Rodgers JB Toth IE Boissonneault GA Bruckner GG Influence of dietary protein and lipid on weight loss in obese ovariohysterectomized cats Am J Vet Res 2000 56 559 565 10.2460/ajvr.2000.61.559 10803653

[B26] Pazak HE Bartges JW Cornelius LC Scott MA Gross K Huber TL Characterization of serum lipoprotein profiles of healthy, adult cats and idiopathic feline hepatic lipidosis patients J Nutr 1998 56 2747S 2750S 9868255 10.1093/jn/128.12.2747S

[B27] Giannini EG Testa R Savarino V Liver enzyme alteration: A guide for clinicians CMAJ 2005 56 367 379 10.1503/cmaj.1040752 15684121 PMC545762

[B28] Mazaki-Tovi M Abood SK Segev G Schenck PA Alterations in adipokines in feline hepatic lipidosis J Vet Intern Med 2013 56 242 249 10.1111/jvim.12055 23480841

[B29] El-Badry AM Graf R Clavien P-A Omega 3 – Omega 6: What is right for the liver? J Hepatol 2007 56 718 725 10.1016/j.jhep.2007.08.005 17869370

[B30] Ibrahim WH Szabo J Sunvold GD Kelleher JK Bruckner GG Effect of dietary protein quality and fatty acid composition on plasma lipoprotein concentrations and hepatic triglyceride fatty acid synthesis in obese cats undergoing rapid weight loss Am J Vet Res 2000 56 566 572 10.2460/ajvr.2000.61.566 10803654

[B31] Hynes AMJ Rouvinen-Watt K Monitoring blood glucose levels in female mink during the reproductive cycle: 1. Prevention of hyperglycemia during the nursing period Can J Vet Res 2007 56 241 248 17955897 PMC1940270

[B32] Hsu WH Hembrough FB Intravenous glucose tolerance test in cats: influenced by acetylpromazine, ketamine, morphine, thiopental, and xylazine Am J Vet Res 1982 56 2060 2061 7181206

[B33] Wamberg S Clausen TN Olesen CR Hansen O Nursing sickness in lactating mink (*Mustela vison*) II. Pathophysiology and changes in body fluid composition Can J Vet Res 1992 56 95 101 1591662 PMC1263515

[B34] Tauson A-H Forsberg M Chwalibog A Plasma concentrations of leptin mirror changes in body weight but do not influence the pattern of preovulatory luteinizing hormone surge in mink (*Mustela vison*) J Nutr 2002 56 1790S 1792S 12042530 10.1093/jn/132.6.1790S

[B35] Ahlstrøm Ø Skrede A Liver fatty acid composition and peroxisomal fatty acid oxidase activity in blue foxes (*Alopex lagopus*) and mink (*Mustela vison*) fed diets containing different levels of fish oil Comp Biochem Physiol A 1997 56 135 140 10.1016/S0300-9629(96)00287-3 9185341

[B36] Yang Z-H Miyahara H Mori T Doisaki N Hatanaka A Beneficial effects of dietary fish-oil-derived monounsaturated fatty acids on metabolic syndrome risk factors and insulin resistance in mice J Agric Food Chem 2011 56 7482 7489 10.1021/jf201496h 21627145

