# Supplementary material for: High feeding intensity increases the severity of fatty liver in the American mink (Neovison vison) with potential ameliorating role for long-chain n-3 polyunsaturated fatty acids
Source: Acta Vet Scand. 2014 Jan 16;56(1):5. doi: 10.1186/1751-0147-56-5 (PMC3896742; doi:10.1186/1751-0147-56-5)
Supplement: Additional file 1 — P-values of the main effects and interactions for mink body weights, feed intake, and blood glucose during the feeding trial from September to November. [file 1751-0147-56-5-S1.docx]

**Additional file 1** P-values of the main effects and interactions for mink body weights, feed intake, and blood glucose during the feeding trial from September to November.

| Effect | Body Weights, Feeding Trial, g | Body Weights, Fasted Mink, g | Feed Intake, *kJ/day* | Feed Intake, *DM g/day* | Feed Intake, *% RDA* | Blood Glucose, mmol/L |
| --- | --- | --- | --- | --- | --- | --- |
| Sex | <0.001 | 0.001 | <0.001 | <0.001 | 0.46 | 0.76 |
| Diet | 0.31 | 0.91 | 0.002 | 0.64 | 0.68 | 0.51 |
| Sex×Diet | 0.59 | 0.43 | 0.25 | 0.35 | 0.74 | 0.82 |
| FI | <0.001 | <0.001 | <0.001 | <0.001 | <0.001 | 0.29 |
| Sex×FI | 0.27 | 0.018 | 0.06 | 0.06 | 0.48 | 0.95 |
| Diet×FI | 1.00 | 0.88 | 0.84 | 0.74 | 0.66 | 0.84 |
| Sex×Diet×FI | 0.86 | 0.90 | 0.71 | 0.68 | 0.72 | 0.32 |
| Time | <0.001 | <0.001 | <0.001 | 0.57 | <0.001 | <0.001 |
| Sex×Time | <0.001 | <0.001 | 0.51 | 0.90 | 0.58 | 0.49 |
| Diet×Time | 0.24 | 0.42 | 0.001 | 0.72 | 0.38 | 0.27 |
| Sex×Diet×Time | 0.17 | 0.71 | 0.89 | 0.95 | 0.26 | 0.44 |
| FI×Time | <0.001 | 0.002 | <0.001 | <0.001 | <0.001 | 0.11 |
| Sex×FI×Time | <0.001 | 0.80 | 0.85 | 0.97 | 0.61 | 0.79 |
| Diet×FI×Time | 0.25 | 0.82 | 0.30 | 0.50 | 0.41 | 0.37 |
| Sex×Diet×FI×Time | 0.55 | 0.23 | 0.54 | 0.52 | 0.24 | 0.70 |

Dry matter, DM; feeding intensity, FI.
