# Supplementary material for: High feeding intensity increases the severity of fatty liver in the American mink (Neovison vison) with potential ameliorating role for long-chain n-3 polyunsaturated fatty acids
Source: Acta Vet Scand. 2014 Jan 16;56(1):5. doi: 10.1186/1751-0147-56-5 (PMC3896742; doi:10.1186/1751-0147-56-5)
Supplement: Additional file 2 — P-values of the main effects and interactions for final body weight and liver responses. [file 1751-0147-56-5-S2.docx]

**Additional file 2** P-values of the main effects and interactions for final body weight and liver responses.

| Effect | Final Body Weight, g | Liver Lipids, % | Liver Weight, g | HSI |
| --- | --- | --- | --- | --- |
| Fast | <0.001 | <0.001 | 0.95 | <0.001 |
| Sex | 0.001 | 0.56 | 0.001 | 0.013 |
| Fast×Sex | 0.22 | 0.64 | 0.79 | 0.26 |
| Diet | 0.12 | 0.33 | 0.18 | 0.58 |
| Fast×Diet | 0.27 | 0.98 | 0.41 | 0.97 |
| Sex×Diet | 0.28 | 0.68 | 0.72 | 0.69 |
| Fast×Sex×Diet | 0.009 | 0.64 | 0.023 | 0.86 |
| FI | <0.001 | 0.004 | <0.001 | 0.19 |
| Fast×FI | 0.78 | 0.020 | 0.07 | 0.44 |
| Sex×FI | 0.004 | 0.19 | 0.005 | 0.39 |
| Fast×Sex×FI | 0.79 | 0.12 | 0.35 | 0.67 |
| Diet×FI | 0.72 | 0.031 | 0.033 | 0.020 |
| Fast×Diet×FI | 0.96 | 0.08 | 0.45 | 0.63 |
| Sex×Diet×FI | 0.52 | 0.85 | 0.65 | 0.47 |
| Fast×Sex×Diet×FI | 0.90 | 0.96 | 0.41 | 0.16 |

Feeding intensity, FI; hepato-somatic index, HSI.
