# Supplementary material for: High feeding intensity increases the severity of fatty liver in the American mink (Neovison vison) with potential ameliorating role for long-chain n-3 polyunsaturated fatty acids
Source: Acta Vet Scand. 2014 Jan 16;56(1):5. doi: 10.1186/1751-0147-56-5 (PMC3896742; doi:10.1186/1751-0147-56-5)
Supplement: Additional file 3 — P-values of the main effects and interactions for the mink clinical chemistry and endocrinological responses. [file 1751-0147-56-5-S3.docx]

**Additional file 3** P-values of the main effects and interactions for the mink clinical chemistry and endocrinological responses.

| Effect | Plasma Glucose, mmol/L | ALT, U/L | AKPH, U/L | Creatine Kinase, U/L | LDL-Cholesterol, mmol/L | HDL-Cholesterol, mmol/L | TAG, mmol/L | FFA, mmol/L | Insulin, µU/mL | Leptin ng/mL | T_3_, nmol/L | T_4_, nmol/L |
| --- | --- | --- | --- | --- | --- | --- | --- | --- | --- | --- | --- | --- |
| Fast | <0.001 | <0.001 | 0.57 | <0.001 | <0.001 | 0.004 | <0.001 | <0.001 | 0.003 | <0.001 | <0.001 | <0.001 |
| Sex | 0.15 | 0.75 | 0.62 | 0.70 | 0.52 | 0.022 | 0.039 | 0.31 | 0.030 | 0.06 | 0.045 | 0.75 |
| Fast×Sex | 0.49 | 0.28 | 0.98 | 0.43 | 0.44 | 0.48 | 0.34 | 0.70 | 0.20 | 0.43 | 0.81 | 0.37 |
| Diet | 0.38 | 0.22 | 0.76 | 0.55 | 0.75 | 0.004 | 0.05 | 0.30 | 0.70 | 0.11 | 0.82 | 0.68 |
| Fast×Diet | 0.76 | 0.94 | 0.78 | 0.26 | 0.61 | 0.65 | 0.17 | 0.53 | 0.30 | 0.63 | 0.009 | 0.08 |
| Sex×Diet | 0.57 | 0.85 | 0.90 | 0.85 | 0.042 | 0.035 | 0.39 | 0.83 | 0.55 | 0.42 | 0.48 | 0.81 |
| Fast×Sex×Diet | 0.29 | 0.95 | 0.23 | 0.52 | 0.37 | 0.36 | 0.23 | 0.49 | 0.58 | 0.27 | 0.029 | 0.60 |
| FI | 0.19 | 0.91 | 0.043 | 0.23 | 0.002 | 0.001 | 0.17 | 0.58 | 0.044 | <0.001 | <0.001 | 1.00 |
| Fast×FI | 0.050 | 0.26 | 0.047 | 0.09 | 0.026 | 0.23 | 0.28 | 0.40 | 0.24 | <0.001 | 0.85 | 0.11 |
| Sex×FI | 0.65 | 0.46 | 0.59 | 0.85 | 0.17 | 0.68 | 0.95 | 0.94 | 0.16 | 0.67 | 0.29 | 0.38 |
| Fast×Sex×FI | 0.17 | 0.28 | 0.98 | 0.11 | 0.43 | 0.17 | 0.95 | 0.68 | 0.51 | 0.24 | 0.39 | 0.14 |
| Diet×FI | 0.57 | 0.52 | 0.97 | 0.58 | 0.07 | 0.002 | 0.51 | 0.015 | 0.60 | 0.33 | 0.32 | 0.021 |
| Fast×Diet×FI | 0.94 | 0.06 | 0.43 | 0.82 | 0.37 | 0.80 | 0.042 | 0.016 | 0.35 | 0.95 | 0.40 | 0.14 |
| Sex×Diet×FI | 0.10 | 0.23 | 0.88 | 0.15 | 0.64 | 0.25 | 0.49 | 0.45 | 0.55 | 0.80 | 0.009 | 0.23 |
| Fast×Sex×Diet×FI | 0.22 | 0.38 | 0.67 | 1.00 | 0.56 | 0.46 | 0.14 | 0.32 | 0.23 | 0.27 | 0.33 | 0.65 |

Alanine transaminase, ALT; alkaline phosphatase, AKPH; feeding intensity, FI; free fatty acid, FFA; high-density lipoprotein, HDL; low-density lipoprotein, LDL; triacylglycerols, TAG; thyroxine, T_4_; triiodothyronine, T_3_.
